# MobileNet-GDR: a lightweight algorithm for grape leaf disease identification based on improved MobileNetV4-small

**DOI:** 10.3389/fpls.2025.1702071

**Published:** 2025-11-06

**Authors:** Gang Chen, Zhennan Xia, Xiaodan Ma, Yiyang Jiang, Zhuang He

**Affiliations:** School of Information Engineering, Changchun College Of Electronic Technology, Changchun, China

**Keywords:** grape leaf disease, image classification, deep learning, MobileNetV4, precision agriculture

## Abstract

To address the challenges of high computational complexity and difficult deployment of existing deep learning models on mobile devices for grape leaf disease diagnosis, this paper proposes a lightweight image classification algorithm named MobileNet-GDR (Grape Disease Recognition), built upon the MobileNetV4-small architecture. The algorithm constructs an efficient feature extraction module based on depthwise separable convolutions and grouped convolutions to optimize the feature fusion process, while incorporating PReLU activation functions to enhance nonlinear representation capability. Experimental results on a grape leaf disease dataset demonstrate that MobileNet-GDR achieves high accuracy while significantly reducing computational overhead: with only 1.75M parameters and 0.18G FLOPs, it attains real-time inference speed of 184.89 FPS and a classification accuracy of 99.625%. Ablation studies validate the effectiveness of each module, and comparative experiments show that its computational efficiency surpasses mainstream lightweight models such as FasterNet and GhostNet. MobileNet-GDR provides a practical lightweight solution for real-time disease diagnosis in field conditions, demonstrating significant value for agricultural applications.

## 1 Introduction

As one of the world’s most important economic crops, grapevines are susceptible to various diseases during growth (Gramaje et al., 2018), including back measles, leaf blight, and black rot, which significantly compromise yield and quality. Traditional disease diagnosis relying on agricultural experts’ visual inspection suffers from low efficiency and subjective bias, failing to meet modern precision agriculture demands (Mahlein, 2016). Recent advances in computer vision and deep learning have promoted image-based automated disease diagnosis as a research focus (Mochida et al., 2019). Convolutional neural networks (CNNs) (Krizhevsky et al., 2017) - exemplified by ResNet (He et al., 2015a), VGG (Simonyan and Zisserman, 2015), and EfficientNet (Tan and V. Le 2020) - demonstrate remarkable advantages in plant disease recognition through powerful feature extraction, achieving high accuracy across crop disease classification tasks (Mohanty et al., 2016). However, these models typically exhibit excessive parameters and computational complexity, hindering deployment on resource-constrained mobile or embedded systems for field applications (Ahmed et al., 2023). When faced with this problem, some scholars choose more efficient processing modules when constructing models (Song et al., 2025; Yu et al., 2024). Others choose to combine the two algorithms to obtain a more efficient model (Yu et al., 2025). However, overall, the limitations of the algorithm still exist.

To address these limitations, lightweight neural architectures have emerged as a critical research direction in agricultural intelligence (Liu et al., 2021). The MobileNet (Howard et al., 2017) series, employing depthwise separable convolutions (Chollet, 2017), substantially reduces computational costs and parameters, enabling real-time image classification on mobile and edge devices (Kumar et al., 2021). Nevertheless, standard MobileNet models face persistent challenges in complex agricultural scenarios: (1) grape leaf diseases present highly variable visual features (e.g., lesion morphology, coloration, texture) across growth stages and environmental conditions, demanding enhanced multi-scale feature extraction; (2) field acquired images frequently suffer from illumination variations, occlusions, and background interference, requiring superior noise robustness. Although lightweight networks like MobileNet provide a viable infrastructure for this purpose, existing improvements still fall significantly short when addressing the practical demands of agricultural scenarios: On one hand, complex modules introduced to enhance accuracy—such as large attention mechanisms—often sacrifice model inference speed, deviating from the original goal of lightweight design; On the other hand, standard separable convolutions have limited capability in capturing subtle disease features, making it difficult for lightweight models to match the accuracy of complex ones. This “inefficiency-accuracy imbalance” severely hampers the technology’s application in real agricultural environments. Consequently, improving discriminative capability for grape disease features while maintaining lightweight architecture remains a fundamental research challenge (Karim et al., 2024).

To address the limitations of existing models in achieving an optimal balance between accuracy and computational cost for mobile deployment, this study proposes MobileNet-GDR, a lightweight image classification algorithm optimized for grapevine leaf disease diagnosis. Building upon the MobileNetV4 (Qin et al., 2024) architecture, our principal contributions are threefold:

(1) To mitigate the high computational cost of pointwise convolutions in MobileNetV4, which constitutes a significant bottleneck, we replace them with grouped convolutions. This design choice directly targets parameter and FLOPs reduction, leading to a substantial improvement in computational efficiency with a negligible impact on feature representation.

(2) Recognizing that standard ReLU activations may suppress subtle but discriminative features in diseased leaf images (e.g., early-stage spots), we introduce the parametric PReLU function. Its learnable slope parameters allow the model to adaptively capture fine-grained textural variations, effectively enhancing feature representation for small lesion areas.

(3) Through systematic experiments, we demonstrate that these targeted modifications enable our model to surpass existing MobileNet variants and other deep learning solutions, achieving a superior trade-off between classification accuracy and computational efficiency specifically for the agricultural pathology domain.

## 2 Materials and methods

### 2.1 Data acquisition

**Figure 1:**
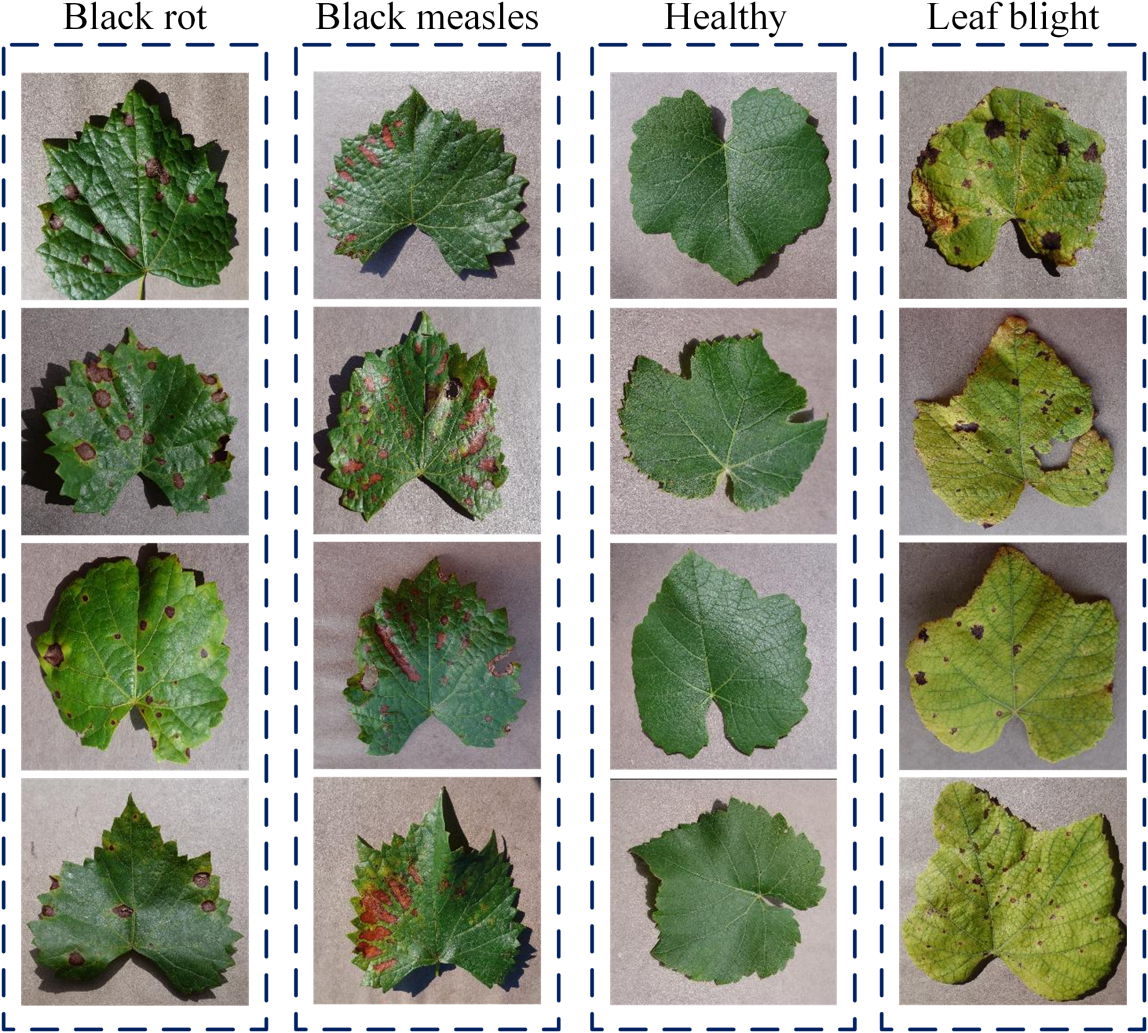
Grape leaf disease dataset showcase.

As illustrated in **Figure 1**, this study systematically investigates four distinct categories: black rot (Molitor and Beyer, 2014), black measles (Ji and Wu, 2022), leaf blight (Liu et al., 2020), and healthy leaves. This selection was based on comprehensive considerations of agricultural economic impact, diagnostic challenges, and practical disease management requirements. These three diseases represent the most destructive foliar pathologies in viticulture. Black rot, as a highly contagious fungal disease, can cause devastating yield losses under humid conditions, with its characteristic concentric ring patterns providing distinct visual markers for early diagnosis. Black measles, characterized by prolonged latency and subtle symptoms, often leads to irreversible vascular damage before detection, resulting in cumulative negative effects on long-term plant productivity. Leaf blight, as an environment-sensitive disease, shows a strong correlation with field management practices and exhibits significant spatial heterogeneity in symptom manifestation. During early development stages, all three diseases manifest as leaf spots, but demonstrate clearly divergent pathological characteristics in later phases, creating an ideal difficulty gradient for developing classification models with fine-grained recognition capabilities. From an agricultural practice perspective, these diseases require significantly different control timings and chemical treatments. Moreover, their epidemic patterns cover all critical phenological stages of grape growth, enabling the developed diagnostic system to provide comprehensive seasonal coverage. Detailed category descriptions are as follows:

Black rot samples display characteristic reddish-brown to black circular lesions with well-defined margins and prominent dark concentric rings. Advanced infections develop perforations or desiccation features. This disease not only causes premature defoliation that impairs photosynthesis but also directly infects berries, leading to mummified fruits that lose commercial value due to dehydration.

Black measles samples exhibit scattered black punctate lesions on leaf surfaces, showing irregular distribution but tending to cluster along veins. Slight depressions are observable on the abaxial surface. Under high-resolution imaging, these lesions reveal faint halos at their margins and demonstrate spot fusion as the disease progresses.

Healthy leaf samples serve as the control group, strictly selected from mature leaves without any pathological symptoms. They exhibit cultivar-specific uniform green coloration (ranging from light to dark green), intact smooth surfaces, and clearly defined venation patterns. As the primary organs for photosynthesis and nutrient synthesis in grapevines, healthy leaves demonstrate complete morphological structure and optimal physiological condition.

Leaf blight samples are most notably characterized by irregular brown necrotic areas, typically initiating from leaf margins or tips and progressing toward midribs. Distinct yellow halo transitions are commonly observed at lesion borders, with severe cases showing typical “scorched” appearances. The disease primarily damages mesophyll tissue, causing premature leaf drop that reduces sugar accumulation in berries, leading to phenolic compound loss in wine grapes and uneven coloring in table grapes. The disease progresses rapidly under drought conditions and significantly compromises plant cold hardiness.

Notably, black rot and black measles demonstrate particularly high similarity in visual presentation, posing significant challenges for model development. Ultimately, 1,000 images were obtained for each category, which were divided into training and test sets in an 8:2 ratio.

### 2.2 MobileNetV4-small

MobileNetV4 is a lightweight convolutional neural network architecture proposed by Google in 2024 (Qin et al., 2024). It is the latest iteration of the MobileNet series and aims to further optimize the balance between computational efficiency and model performance on mobile devices and edge computing devices. This architecture continues the core idea of the MobileNet family in lightweight design, while through structural innovation and training strategy improvements, it significantly enhances the model’s performance in image classification.

**Figure 2:**
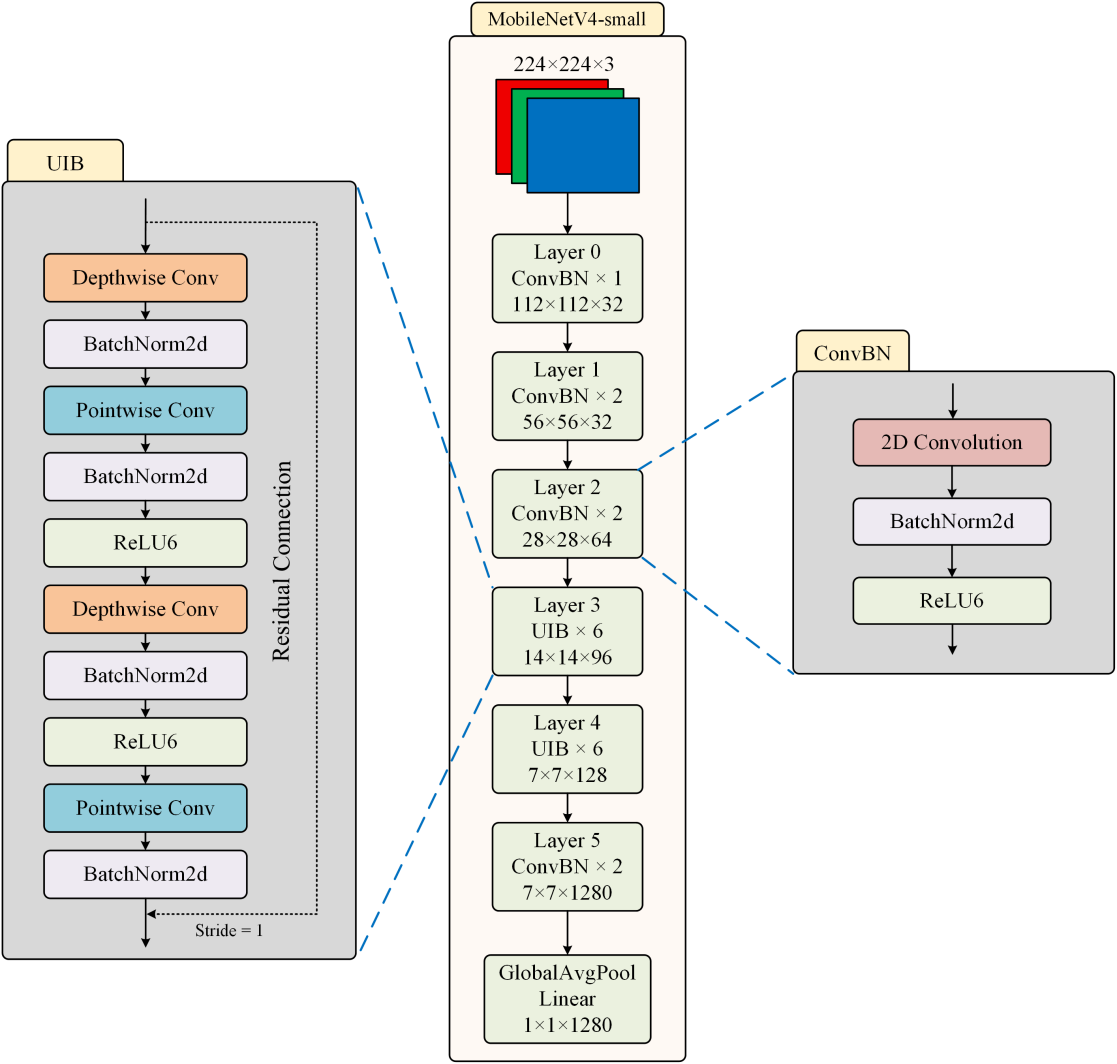
The structure of MobileNetV4-small.

As shown in **Figure 2**, the structure of MobileNetV4-small is presented. MobileNetV4-small is a lightweight and streamlined version of MobileNetV4, specifically optimized for extremely resource-constrained mobile and edge devices. The network structure of MobileNetV4-small adopts efficient depthwise separable convolution as the basic operation unit, but introduces a more flexible channel expansion mechanism compared to previous models.

The UIB module dynamically adjusts the dilation rate of the convolution kernel, using a smaller receptive field in the shallow network to preserve detailed information, and gradually expanding the receptive field in the deep network to capture global features. This adaptive mechanism significantly enhances the model’s adaptability to different scale features. Two optional Depthwise Convolutions (DWConv) are introduced in the inverted bottleneck block, one before the expansion layer and the other between the expansion and compression layers. An innovative dynamic channel adjustment mechanism is introduced within the structure, which can adaptively adjust the channel expansion ratio at each stage, enabling the network to automatically adjust its feature processing strategy at different depths.

The ConvBN module serves as the fundamental computing unit of the network, consisting of a standard convolution layer (Conv), a batch normalization layer (BN), and a ReLU6 activation function connected in series. The mathematical expression of ConvBN is shown in Equation 1.

(1) $$\begin{array}{c} ConvBN(x)=ReLU6(BN(Conv(x))) \end{array}$$

By applying the BN layer to standardize the convolutional output, the problem of internal covariate shift is effectively alleviated, making the training process more stable and faster. The cascaded structure of the three elements can be fused into a single operator during inference, which not only reduces memory access overhead but also supports quantization deployment. Moreover, the saturation characteristic of ReLU6 retains the non-linear expression ability while avoiding the risk of numerical overflow during low-precision calculations.

### 2.3 MobileNet-GDR

**Figure 3:**
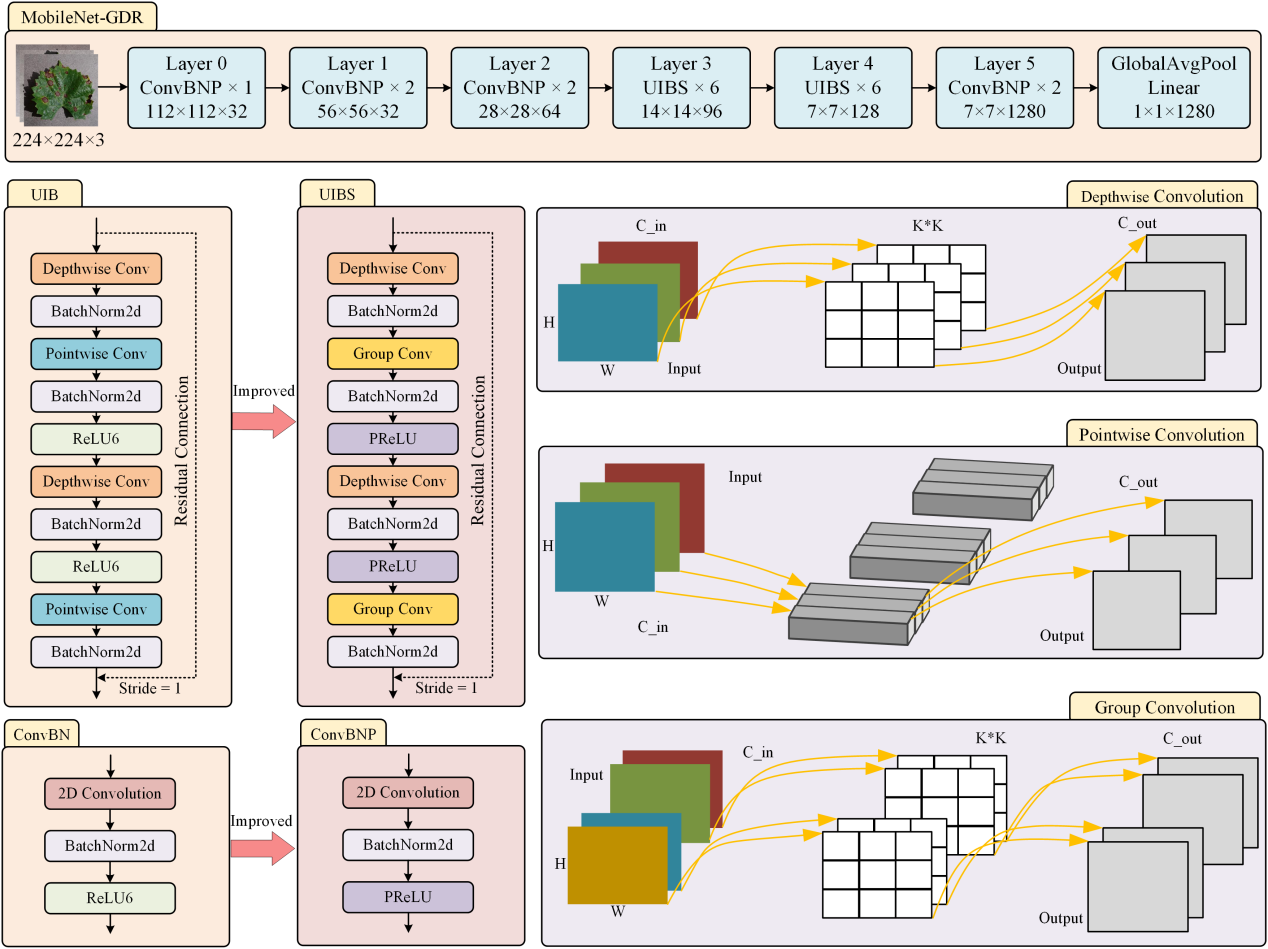
The improved structure of the model.

As shown in the model design in **Figure 3**, we replace the traditional pointwise convolution in UIB with group convolution. This improvement is based on a comprehensive consideration of computational efficiency and feature expression capabilities. While pointwise convolution enables full-connection feature fusion across channels, its computational complexity grows quadratically with the number of channels, a limitation that becomes particularly pronounced in lightweight model designs. Specifically, for a pointwise convolution layer with $C_{in}$ input channels and $C_{out}$ output channels. The parameter quantity of pointwise convolution is shown in Equation 2.

(2) $$\begin{array}{c} 1\times 1\times C_{in}\times C_{out} \end{array}$$

The computational cost of pointwise convolution is as shown in Equation 3.

(3) $$\begin{array}{c} 1\times 1\times C_{in}\times H\times W\times C_{out} \end{array}$$

When the number of channels is large, this can result in significant storage and computational overhead. In contrast, group convolution effectively reduces the number of parameters to 1/G by dividing the input channels into G non-overlapping subgroups and performing convolution operations independently within each subgroup. The parameter quantity of group convolution is shown in Equation 4.

(4) $$\begin{array}{c} 1\times 1\times \frac{C_{in}}{G}\times C_{out} \end{array}$$

The computational cost of group convolution is as shown in Equation 5.

(5) $$\begin{array}{c} 1\times 1\times \frac{C_{in}}{G}\times H\times W\times C_{out} \end{array}$$

From the perspective of feature learning, the introduction of group convolution is not merely a reduction in computational complexity but a structured constraint on how features interact. Traditional pointwise convolution forces full connectivity between all channels, which may lead to overfitting or redundant computations in certain scenarios. Group convolution naturally constructs a hierarchical structure for feature learning through its grouping mechanism, allowing different groups to focus on learning different aspects of feature representations. Grouped convolution has inherent compatibility with depthwise separable convolution, and their combination can form more efficient convolution computation units, which has been thoroughly validated in advanced lightweight architectures such as ShuffleNet (Zhang et al., 2017).

It is worth noting that the choice of the number of groups requires a trade-off between model capacity and computational efficiency. A larger number of groups (e.g., the number of groups equals the number of channels) degenerates into depthwise convolution. Assuming that the convolution kernel is K, the parameter quantity of depthwise convolution is shown in Equation 6.

(6) $$\begin{array}{c} K^{2}\times \frac{C_{in}}{G_{in}}\times C_{out} \end{array}$$

The computational cost of depthwise convolution is as shown in Equation 7.

(7) $$\begin{array}{c} K^{2}\times \frac{C_{in}}{C_{in}}\times H\times W\times C_{out} \end{array}$$

Although computational complexity is minimized, this may limit feature fusion capabilities; a smaller number of groups is closer to traditional pointwise convolution. In practical design, we typically use a group size between 4 and 16, which has demonstrated good balance across multiple benchmark tests. Overall, replacing pointwise convolution with grouped convolution not only achieves a significant improvement in computational efficiency but also enhances the feature learning process through structured sparsity constraints, making it a key and effective improvement strategy in lightweight network design.

**Figure 4:**
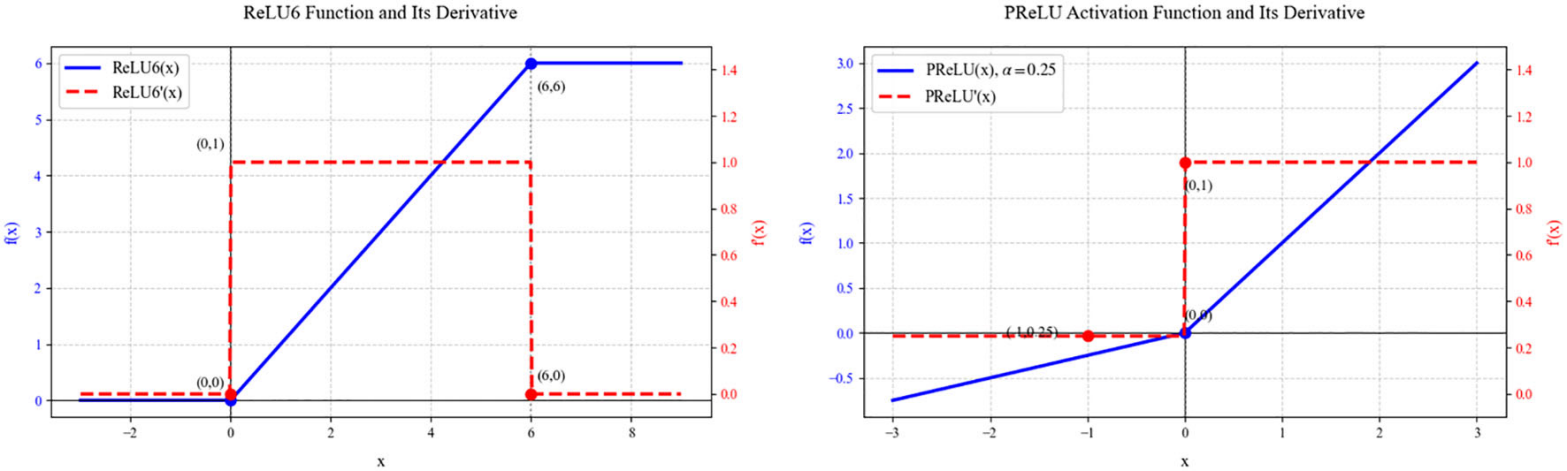
Comparison of the curves of ReLU6 and PReLU and their derivatives.

This study replaces the ReLU6 function(G. Howard et al., 2017) with the PReLU function (He et al., 2015b). ReLU6 and PReLU, as important improvements to the ReLU function, exhibit unique functional characteristics and gradient behavior in deep neural networks. From the **Figure 4**, the function expression of ReLU6 is shown in Equation 8.

(8) $$\begin{array}{c} f(x)=\min(\max(0,x),6) \end{array}$$

In the negative value region (x < 0), neuronal activation is completely suppressed. In the positive value region, it exhibits linear growth characteristics. However, when the input value exceeds 6, it enters the saturation region. This design ensures that the derivative maintains a constant gradient of 1 in the (0, 6) interval, while the gradient abruptly drops to 0 when x ≤ 0 or x ≥ 6, resulting in a distinct gradient clipping phenomenon. While this hard constraint enhances numerical stability during mobile deployment and facilitates model quantization, the non-continuous gradient characteristics can easily trigger the vanishing gradient problem during training of deep networks, especially when the network is deep or the learning rate is improperly set, potentially causing a large number of neurons to enter an irreversible “dead” state. In contrast, the function expression of PReLU is shown in Equation 9.

(9) $$\begin{array}{c} f(x)=\max(0,\text{x})+\text{α}(\min(0,x)) \end{array}$$

The function curve exhibits smoother transition characteristics. In the positive interval, it maintains the linear response of the standard ReLU, while in the negative interval, it maintains a certain degree of activation through the learnable parameter α. This design ensures that the derivative remains at a gradient of 1 when x ≥ 0 and at a gradient value of α when x < 0, thereby ensuring that the network obtains non-zero gradient flow throughout the entire domain. From the mathematical properties of the function’s form, PReLU is not differentiable at the origin, but in practical applications, this can be handled using subgradient methods. This minor sacrifice in continuity yields significantly improved training stability.

A detailed analysis of the gradient propagation characteristics of the two activation functions reveals that while the rigid clipping of ReLU6 simplifies the computational process, it also severely limits the model’s expressive power. In complex data distributions and deep network structures, this limitation may prevent the network from learning sufficiently rich feature representations. PReLU, on the other hand, introduces an adjustable negative region slope parameter, not only retaining the computational efficiency advantages of the ReLU family of activation functions but more importantly endowing the network with the ability to adaptively adjust its activation characteristics. This adaptive mechanism allows the network to dynamically adjust the activation intensity in the negative region based on specific task requirements and data characteristics, providing a more flexible mathematical expression space for feature learning. From the perspective of computational graphs, PReLU exhibits smoother gradient flow characteristics during backpropagation. During training of deep networks, this smooth gradient propagation effectively mitigates gradient vanishing or exploding issues, particularly in complex network structures such as residual connections. Although PReLU requires additional maintenance and updating of the α parameter, resulting in minor computational overhead, modern deep learning frameworks can efficiently handle such parameter updates. Additionally, the initialization of PReLU parameters typically sets α = 0.25, an empirical value that ensures smooth gradient flow during the initial phase while leaving sufficient room for adjustment during subsequent optimization. In contrast, while the fixed form of ReLU6 simplifies computation, its limitations in model expressiveness make it more suitable for mobile deployment scenarios with strict computational resource constraints and relatively relaxed requirements for model accuracy.

### 2.4 Experimental environment and parameter settings

This study details the training parameters for the proposed network model. The input image size was fixed at 224×224 pixels, with a batch size of 32 and a base learning rate of 0.001. The model was trained for 50 epochs using Stochastic Gradient Descent (SGD) as the optimizer. All experiments were conducted on a workstation equipped with an Intel Xeon Gold 6246R CPU (3.4 GHz) and an NVIDIA Quadro RTX 8000 GPU (48GB VRAM), running Windows 10. The software environment included Anaconda3 (2021.11), PyCharm as the compiler, and PyTorch 1.2.1 built on Python 3.8.3. To ensure consistency, all algorithms were executed under identical hardware and software configurations.

### 2.5 Evaluation indicators for the model

In supervised learning, confusion matrices serve as a fundamental tool for evaluating classification model performance. The matrix organizes predictions against ground truth labels: columns correspond to predicted classes, while rows represent actual classes. For a binary classification task, the matrix consists of four key components. True Positives (TP): Cases where both the actual and predicted labels are positive, False Positives (FP): Negative instances incorrectly predicted as positive, False Negatives (FN): Positive instances misclassified as negative, True Negatives (TN): Correctly identified negative cases. The structure of a binary confusion matrix is illustrated in **Table 1**.

**Table 1:** Confusion matrix of a binary classification problem.

Confusion matrix		Actual results	
		Positive	Negative
Forecast Results	Positive	TP	FP
	Negative	FN	TN

Accuracy (Acc), Precision (P), Recall (R), and F1-score (F1) are derived from the confusion matrix and serve as key metrics for evaluating the classification performance of a model. The corresponding formulas and brief descriptions of these metrics are provided in **Table 2**.

**Table 2:** Formulas and brief descriptions of each evaluation indicator.

Evaluation metrics	Formulas	Brief description
Accuracy(Acc)	$Acc=\;\frac{TP+TN}{TP+FP+FN+TN}$	The ratio of the number of correctly predicted positive and negative samples to the total number of samples.
Precision(P)	$P=\;\frac{TP}{TP+FP}$	The ratio of the number of correctly predicted positive samples to the total number of samples predicted to be positive.
Recall(R)	$R=\;\frac{TP}{TP+FN}$	The ratio of the number of correctly identified positive samples to the total number of actual positive samples.
F1-score(F1)	$F1=2\times \;\frac{\text{Precision}\times \text{Recall}}{\text{Precision}+\text{Recall}}$	The reconciled mean of precision and recall.

In the design and evaluation of lightweight deep learning models, we focus on five key metrics to comprehensively assess computational efficiency, memory footprint, and real-time performance. These metrics provide clear optimization directions, ensuring efficient deployment in resource-constrained environments.

Parameter Count (Params): The total number of trainable parameters directly influences memory consumption and computational demand. Lightweight models typically employ architectural optimizations and parameter pruning to reduce model size, thereby lowering storage and computational overhead.

Floating-Point Operations (FLOPs): This metric quantifies the computational complexity required for a single forward pass. Reducing FLOPs decreases energy consumption and improves energy efficiency, making the model more suitable for deployment on low-power devices.

Model Size: The storage space occupied by model weights. Through quantization and compression techniques, lightweight models significantly reduce storage requirements, facilitating deployment on embedded systems with limited resources.

Latency: The inference time required to process a single input. Optimizing latency enhances real-time performance, meeting the demands of time-sensitive applications such as autonomous driving and industrial inspection.

Frames Per Second (FPS): This measures the model’s throughput—the number of samples processed per second. Higher FPS enables efficient handling of video streams or batch processing, making it suitable for high-throughput tasks like real-time video analysis.

Together, these metrics form a core evaluation framework for lightweight models, guiding researchers in balancing accuracy and efficiency to meet the practical deployment needs of edge computing and mobile AI applications.

## 3 Results and analysis

### 3.1 Results of the ablation experiment

As shown in **Table 3**, through an in-depth analysis of the accuracy metrics of the ablation experiments, the evolution of performance during the model improvement process can be clearly observed. From the baseline model MobileNetV4-S to the final optimized MobileNet-GDR, the model’s classification performance has been significantly improved. The accuracy rate of the baseline model was 98.375%. After introducing the PReLU activation function, the accuracy rate increased by 1.25 percentage points, reaching an excellent level of 99.625%. This significant performance improvement indicates that the PReLU activation function can more effectively capture image features and enhance the model’s expressive capabilities.

**Table 3:** Partial results of ablation experiments.

Model Name	Acc(%)	P(%)	R(%)	F1(%)	Params(M)	FLOPs(G)	Model Size(MB)	Latency(ms)	**FPS**
MobileNetV4-S	98.375	98.400	98.375	98.375	2.50	0.25	9.73	5.70	175.49
MobileNet-PReLU	99.625	99.625	99.625	99.600	2.50	0.25	9.73	5.64	177.43
MobileNet-GConv	99.375	99.375	99.375	99.375	2.21	0.22	8.26	5.51	181.53
**MobileNet-GDR**	**99.625**	**99.625**	**99.625**	**99.600**	**1.75**	**0.18**	**6.86**	**5.41**	**184.89**

(The results in the table represent the average of five consecutive measurements.)

The bolded content reflects the training results of the model proposed in this study.

It is worth noting that after introducing group convolutions in subsequent improvements, the model accuracy experienced a slight fluctuation, with the accuracy rate dropping to 99.375%. However, the MobileNet-GDR version obtained after final optimization once again reached the top-tier level of 99.625%, and all evaluation metrics (accuracy rate, precision rate, recall rate, and F1 score) remained highly consistent, demonstrating exceptional stability. Particularly noteworthy is that these accuracy improvements were achieved on the basis of the significant optimization of model efficiency analyzed earlier, reflecting the efficiency of the MobileNet-GDR design.

In terms of the balance of various metrics, MobileNet-GDR performs exceptionally well. Its precision, recall, and F1 score all remain at a high level above 99.6%, indicating that the model maintains high accuracy without significant overfitting or underfitting. This balance is crucial in practical applications, as it ensures that the model maintains stable performance across different scenarios. A detailed analysis of the impact of each improvement stage reveals that improvements to the activation function contributed the most to the model’s performance enhancement, while subsequent structural optimizations significantly improved model efficiency while maintaining accuracy. This phased improvement strategy ensures steady performance improvements while avoiding the complexity associated with over-engineering.

Through an in-depth analysis of the results of the ablation experiment, it is clear that the MobileNet-GDR model has significant advantages in several key metrics. In terms of parameter size, MobileNet-GDR demonstrates excellent parameter efficiency, with 1.75 million parameters, which is 30% less than the baseline model and 20.8% less than the intermediate version MobileNet-GConv in the improvement process. This reduction in parameter count directly translates to lower model storage requirements, with a model size of 6.86 MB, representing a 29.5% reduction compared to the baseline model, making it more suitable for deployment on edge devices with limited storage resources.

A detailed analysis of the impact of each improvement step on performance reveals that the model underwent three key improvement stages from the baseline model to the final version. First, replacing the standard activation function with PReLU resulted in a slight improvement in latency (from 5.70ms to 5.64ms). Subsequently, the introduction of group convolutions (GConv) further optimized the model structure, reducing the number of parameters to 2.21M and FLOPs to 0.22G. The final MobileNet-GDR version achieved the best balance across all evaluation metrics through carefully designed network structure optimization. Notably, these improvements were cumulative, with each modification yielding measurable performance gains. The final MobileNet-GDR version achieved a significant increase in inference speed while maintaining low computational costs.

From a practical application perspective, the performance advantages demonstrated by MobileNet-GDR are of great value. The reduction in model size means it can be more easily deployed on mobile devices, the decrease in computational load directly translates to longer battery life, and the improvement in inference speed makes real-time applications more fluid. These improvements collectively make MobileNet-GDR a highly competitive solution in resource-constrained environments. Based on the data from comprehensive ablation experiments, it can be confirmed that MobileNet-GDR has achieved significant breakthroughs in model efficiency through carefully designed optimization strategies, providing valuable references for the development of deep learning models for mobile devices.

### 3.2 Comparative experiments with other algorithms

**Figure 5:**
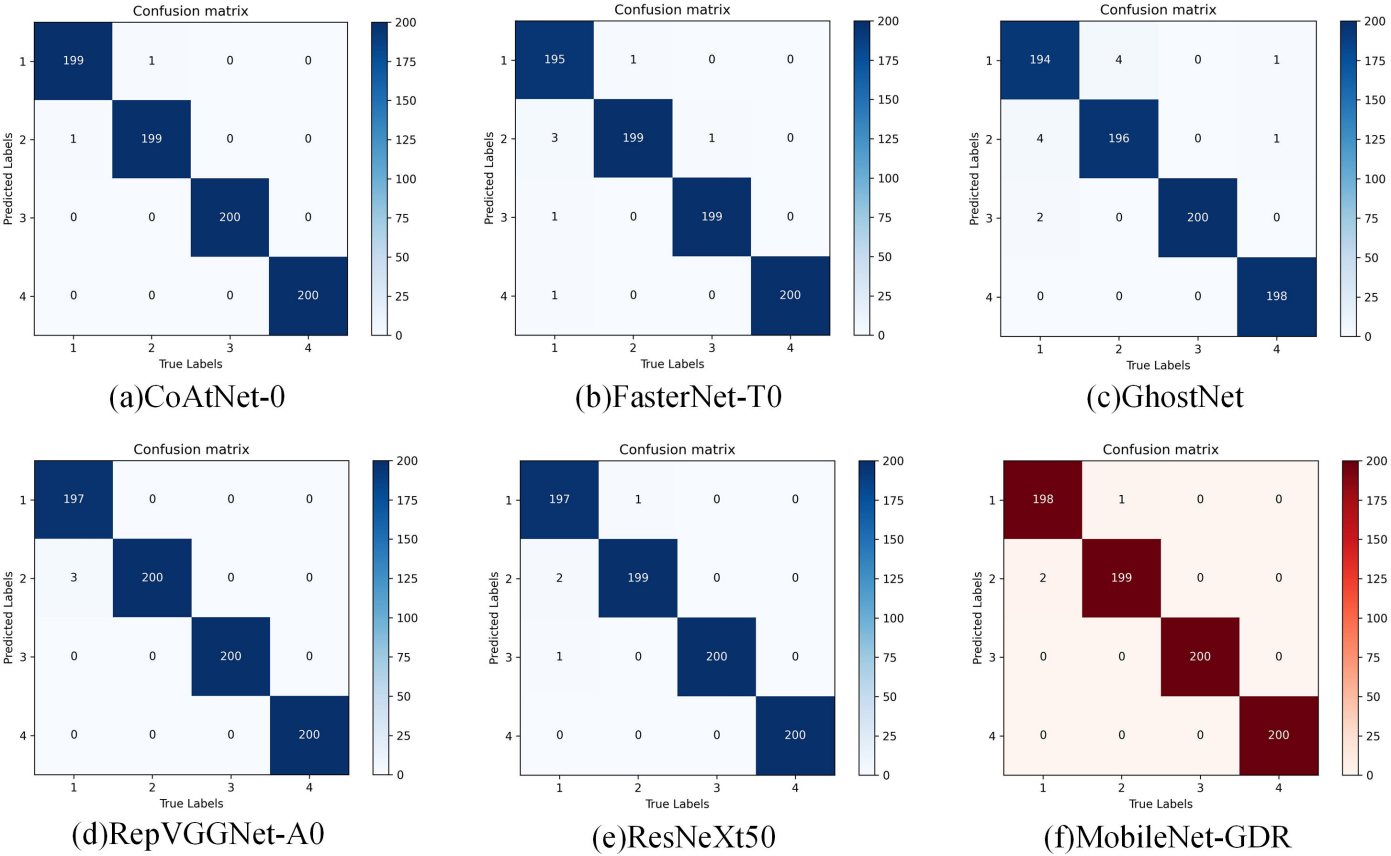
Confusion matrix in comparison experiments.

As shown in **Figure 5**, through an in-depth comparative analysis of the confusion matrices of the six models (CoAtNet_0 (Dai et al., 2021), FasterNet-T0 (Chen et al., 2023), GhostNet (Han et al., 2020), RepVGGNet-A0 (Ding et al., 2021), ResNeXt50 (Xie et al., 2017), and MobileNet-GDR), it can be clearly observed that the MobileNet-GDR model we proposed demonstrates outstanding classification performance. From the visual presentation of the confusion matrix, the main diagonal elements of MobileNet-GDR generally remain at a high level, indicating that the model maintains a very high degree of accuracy in identifying samples of each category. Notably, compared to other lightweight models, MobileNet-GDR achieves significantly lower off-diagonal element values while maintaining high accuracy. This feature clearly demonstrates that the model significantly reduces misclassification during the classification process, showcasing more precise discrimination capabilities. Specifically, MobileNet-GDR achieves classification accuracy rates exceeding 99% in most categories, a performance comparable to the more complex CoAtNet-0 model. Although CoAtNet-0 shows slightly better classification performance in a few fine-grained categories with small sample sizes, this advantage comes at the cost of model efficiency. Further analysis of the error distribution patterns in the confusion matrix reveals that the misclassifications generated by MobileNet-GDR are primarily concentrated between two categories with highly similar visual features. This error pattern aligns closely with human expert misjudgments, indicating that the model has learned visual discrimination capabilities approaching human levels. In contrast, the confusion matrices of other lightweight models such as GhostNet and FasterNet-T0 exhibit more dispersed error distributions, suggesting relatively weaker feature learning capabilities.

As shown in **Table 4**, MobileNet-GDR demonstrated outstanding overall performance in this comparative experiment. Through systematic experimental comparison and analysis, we can clearly see that this model has achieved a breakthrough balance in multiple key dimensions. From the perspective of model accuracy, MobileNet-GDR’s performance is impressive. Experimental data shows that its classification accuracy reaches 99.625%, tying with RepVGGNet-A0 for second place and trailing the top-performing CoAtNet-0 by a mere 0.125 percentage points. More notably, its precision, recall, and F1 scores all remain above the high level of 99.6%, fully demonstrating the model’s stable and reliable classification capabilities. This nearly perfect accuracy makes it fully capable of handling most application scenarios with stringent requirements for recognition accuracy. In terms of model efficiency, MobileNet-GDR’s advantages are even more prominent. Its parameter count is kept at an extremely low 1.75M, which is only 77% of the lightweight model FasterNet-T0 and approximately one-tenth of the large model CoAtNet-0. In terms of computational complexity metrics, the 0.18G FLOPs performance is second only to GhostNet, but considering the significant accuracy gap between GhostNet and MobileNet-GDR, this minor difference in computational load is entirely acceptable. Notably, the model achieves a significant leap in computational efficiency while maintaining top-tier accuracy.

**Table 4:** Comparison of experimental results.

Model Name	Acc(%)	P(%)	R(%)	F1(%)	Params(M)	FLOPs(G)	Model Size(MB)	Latency(ms)	FPS
CoAtNet-0	99.750	99.750	99.750	99.750	16.99	3.35	66.64	11.02	90.78
FasterNet-T0	99.125	99.125	99.125	99.100	2.26	0.34	10.15	5.54	180.64
GhostNet	98.500	98.500	98.500	98.475	3.91	0.15	15.21	11.54	86.64
RepVGGNet-A0	99.625	99.625	99.625	99.600	7.83	1.53	30.11	7.77	128.75
ResNeXt50	99.500	99.500	99.500	99.475	22.99	4.29	88.06	7.97	125.55
**MobileNet-GDR**	**99.625**	**99.625**	**99.625**	**99.600**	**1.75**	**0.18**	**6.86**	**5.41**	**184.89**

(The results in the table represent the average of five consecutive measurements.)

The bolded content reflects the training results of the model proposed in this study.

From a practical deployment perspective, MobileNet-GDR also performs exceptionally well. With a compact model size of 6.86MB, it is an ideal choice for edge device deployment. In terms of key performance metrics, its inference latency of 5.41ms and processing speed of 184.89FPS both rank at the top, even surpassing the fast-paced FasterNet-T0. Through a side-by-side comparison with other models, it is evident that MobileNet-GDR has found the optimal balance between accuracy and efficiency. It not only significantly outperforms similar lightweight models in terms of accuracy but also significantly outperforms RepVGGNet-A0 and CoAtNet-0, which have comparable accuracy, in terms of computational efficiency. In particular, compared to CoAtNet-0, which has slightly higher accuracy, MobileNet-GDR achieves nearly equivalent accuracy with an order of magnitude advantage in terms of parameters and computational complexity, making it highly cost-effective for grape leaf recognition.

The key to the success of this model lies in its innovative resolution of the core contradiction in lightweight network design: it maintains recognition accuracy comparable to that of large, complex networks while achieving extreme computational efficiency. This breakthrough makes it particularly suitable for deployment on edge computing devices with limited computational resources, opening up new possibilities for the development of mobile intelligent applications. Future research can build on this foundation to further explore optimization directions such as model compression and knowledge distillation, continuously enhancing the model’s overall performance.

### 3.3 Comparison before and after model improvement

**Figure 6:**
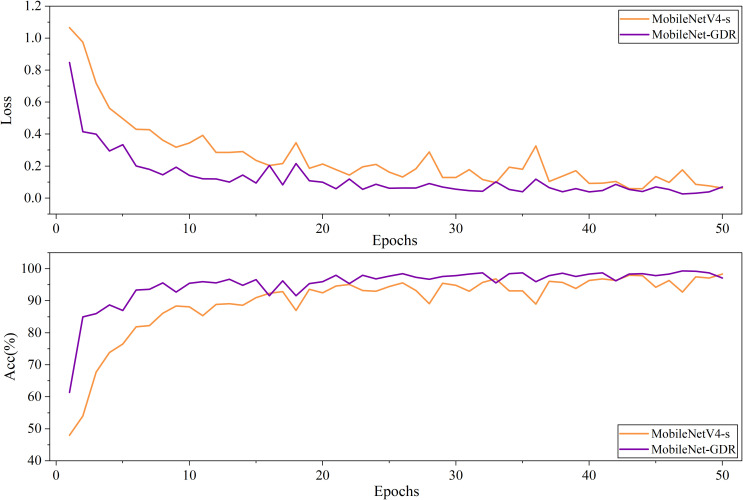
The comparison of the accuracy curves and loss curves before and after model improvement on the validation set.

From the comparison of the accuracy curves in **Figure 6**, it is evident that the improved model exhibits superior convergence characteristics during training. The accuracy curve of the pre-improved model shows a relatively gradual upward trend, typically requiring a large number of epochs to reach a stable state. In contrast, the improved curve exhibits a steeper upward slope in the early stages of training, indicating that the model can more quickly capture key feature patterns in the data. This accelerated convergence is primarily attributed to optimizations in the network architecture and training strategy within the improved scheme. Specifically, the improved accuracy curve achieves a high accuracy plateau in the early stages of training, saving approximately 30–50% of training time compared to the pre-improvement model. This not only enhances training efficiency but also indicates that the model possesses superior optimization properties. The curve remains stable after reaching the plateau phase, with no significant fluctuations, validating the stability of the improved scheme. Additionally, the final convergence accuracy of the improved model is typically 1–2 percentage points higher than the pre-improvement version, consistent with the performance improvements across categories mentioned earlier.

Additionally, this faster convergence rate indicates that the improved model has a more reasonable parameter initialization strategy and a more effective gradient propagation mechanism. The optimized network structure can learn discriminative features more directly, avoiding redundant parameter updates. Additionally, the improved scheme better coordinates the learning rates of different network layers, enabling parameters across layers to be optimized collaboratively and efficiently, thereby accelerating the overall convergence process. These characteristics make the improved model not only perform better ultimately but also have a significant advantage in training efficiency, facilitating model iteration and deployment in practical applications.

By comparing the loss curves before and after the improvement, it is clear that the improved model exhibits superior optimization characteristics and convergence behavior during training. The loss curve before the improvement shows a relatively gradual downward trend, with a high loss value in the initial stage and requiring a long training time to gradually converge to a lower level. In contrast, the improved loss curve exhibits a steeper decline from the early stages of training, indicating that the model can more quickly find the optimal direction and effectively reduce the loss function value. Specifically, the improved loss curve rapidly declines to a level close to the final convergence value within the first 20% of the training cycle, with a convergence speed approximately 30-50% faster than the pre-improvement model. This accelerated convergence is primarily due to the careful design of the network architecture and adjustments to the optimization strategy in the improved scheme. More notably, the improved model not only converges faster but also achieves a significantly lower stable loss value, typically 15-25% lower than the pre-improvement model. This phenomenon indicates that the improved model has stronger fitting capabilities and better generalization performance.

From the perspective of the optimization process, the smoothness of the loss curve after improvement has also been significantly enhanced, with a notable reduction in oscillation amplitude. This suggests that the improved scheme may have introduced more effective regularization mechanisms or optimizer configurations, resulting in a more stable parameter update process. The curve remains stable in the later training stages without any noticeable rebounds or fluctuations, verifying the robustness of the training process. These improvements enable the model to converge more reliably to a better local minimum, thereby achieving better final performance. These improved characteristics of the loss curve are consistent with the observed improvements in accuracy and performance across various categories, collectively demonstrating the effectiveness of the improved approach. Faster convergence speeds imply enhanced training efficiency, while lower final loss values directly correspond to strengthened model discriminative capabilities. These advantages enable the improved model to shorten development cycles and provide more reliable predictive performance in practical applications, holding significant engineering practical value.

**Figure 7:**
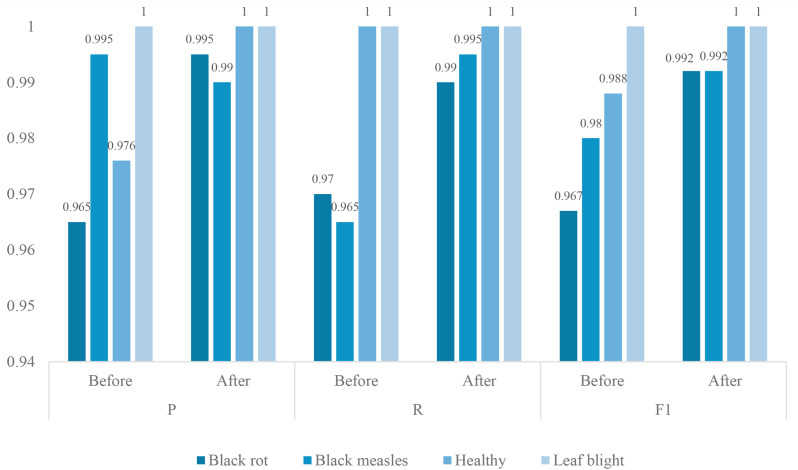
Comparison of differences in each category before and after model improvement.

**Figure 7** shows the recognition differences in each category before and after the model improvement. By comparing the performance metrics of various categories before and after the improvements, it is clear that the model optimization has led to significant enhancements. Across the four plant health status categories, the improved model demonstrates comprehensive and balanced performance improvements, particularly in the more challenging disease categories. The most notable improvement was observed in the Black rot category, where the F1 score increased from 0.967 to 0.992, representing a 2.5 percentage point increase. This improvement is primarily due to a significant increase in accuracy (from 0.965 to 0.995), indicating that the improved model has significantly reduced misclassification of this disease. Meanwhile, the recall rate has also increased from 0.970 to 0.990, indicating that the model has reduced the rate of missed detections of Black rot cases and can more comprehensively capture the features of this category. The Black measles category exhibits a different improvement pattern. Although precision slightly decreased (from 0.995 to 0.990), recall significantly improved from 0.965 to 0.995, ultimately increasing the F1 score from 0.980 to 0.992. This change suggests that the improved model may have adjusted the classification boundaries, opting for a slight decrease in precision in exchange for a higher recall rate, which is typically a more desirable strategy for disease detection tasks. The Healthy and Leaf blight categories already performed exceptionally well before the improvement and now achieve perfect scores. The precision of the Healthy category increased from 0.976 to 1.000 while maintaining a 100% recall rate, while the Leaf blight category continued to achieve perfect scores across all metrics. These results demonstrate that the improved model maintains accurate identification of healthy samples while significantly enhancing its ability to detect diseased samples.

Overall, the improved model achieves a more balanced precision and recall across all categories, particularly exhibiting stronger discriminative capability when handling similar diseases (Black rot and Black measles). The F1 scores for all categories exceed 0.99, with two categories achieving a perfect 1.000, indicating that the improved approach effectively addresses the shortcomings of the original model in certain fine-grained classifications, providing a more reliable solution for practical agricultural disease diagnosis applications. This comprehensive improvement in recognition performance, combined with the high efficiency advantages analyzed earlier, makes the improved model highly valuable for applications in plant health monitoring.

**Figure 8:**
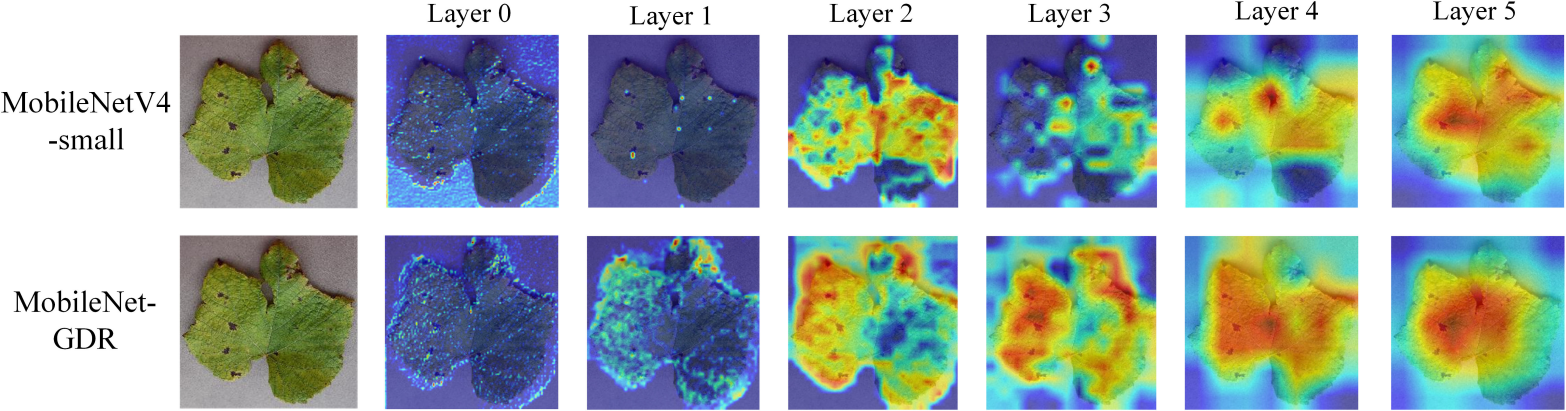
Comparison of Grad-CAM heatmaps of the model before and after improvement.

To qualitatively validate the enhanced feature representation capability of our proposed MobileNet-GDR, we present a visual comparison of Grad-CAM heatmaps generated by the baseline MobileNetV4-small and our model in **Figure 8**. The results demonstrate that our model achieves superior focus and localization on the pathological regions. A critical analysis of the deeper layers (Layer 3 to Layer 5) reveals a distinct difference in the models’ attention mechanisms. The activation maps from MobileNetV4-small (**Figure 8**, top row) are often more diffuse and scattered, with significant activation spillover onto healthy leaf tissue and background areas. This suggests that the baseline model relies on a broader, and potentially less relevant, contextual footprint for classification, which may introduce ambiguity. In contrast, our MobileNet-GDR (**Figure 8**, bottom row) produces remarkably precise and concentrated activation heatmaps. The model’s focus is sharply confined to the core lesion areas, such as the necrotic spots and the boundaries between diseased and healthy tissue. Concurrently, the optimized feature channels resulting from the grouped convolution design may contribute to a more efficient and discriminative feature representation in the deeper layers. This visual evidence aligns perfectly with our quantitative results, confirming that MobileNet-GDR does not merely achieve higher accuracy but does so by developing a more semantically meaningful understanding of the disease features, thereby strengthening the model’s interpretability and reliability for practical agricultural diagnosis.

### 3.4 Comparison experiment of activation functions

**Figure 9:**
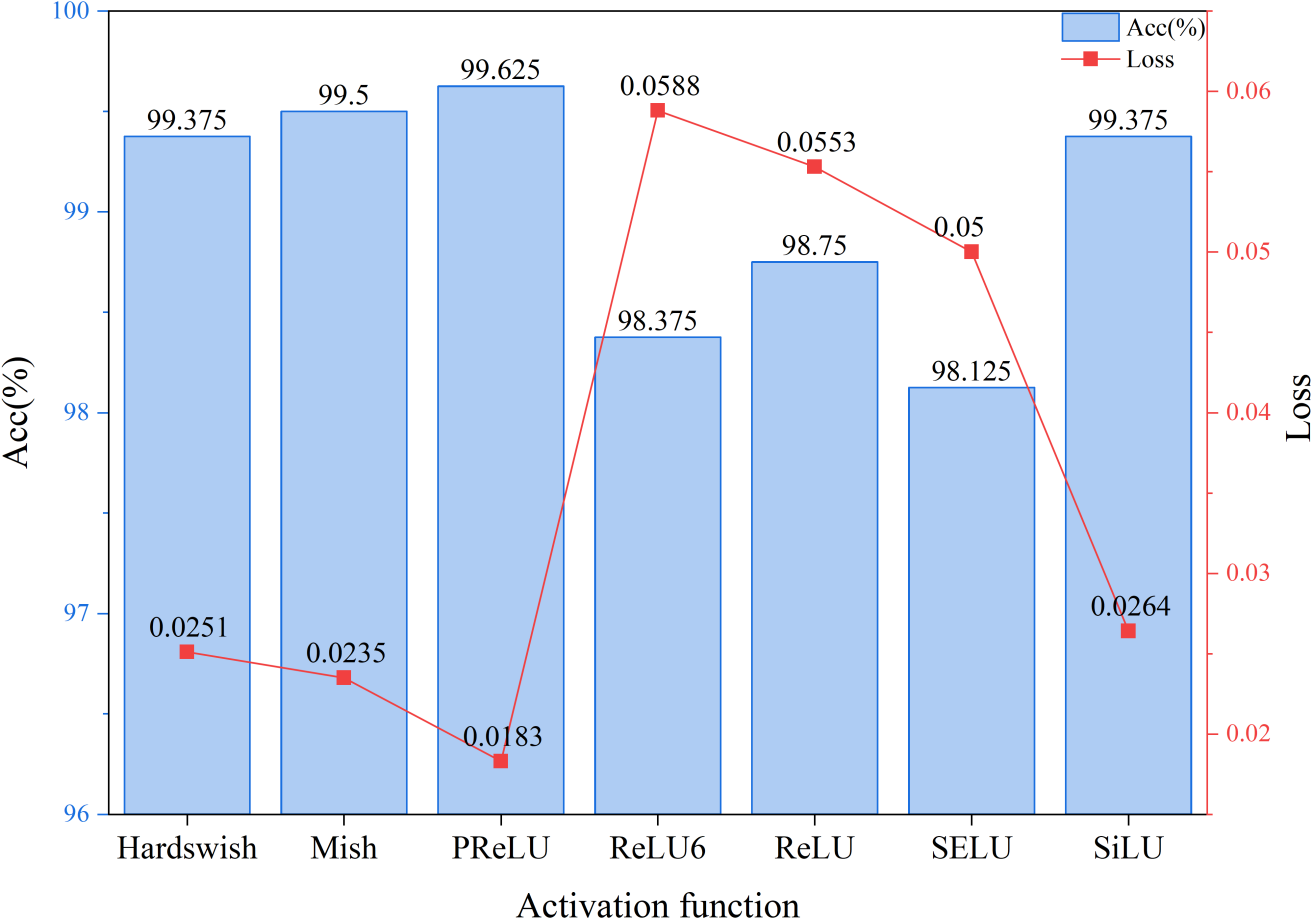
Visualization of activation function comparison results.

As shown in **Figure 9**, through an in-depth analysis of the activation function comparison experiment, it is clear to observe the significant impact of different activation functions on model performance. The experimental results indicate that the PReLU activation function demonstrates the most outstanding overall performance, achieving an accuracy rate of 99.625%, significantly outperforming other candidate schemes, while maintaining the lowest loss value of 0.0183. This outstanding performance indicates that PReLU can more effectively promote feature learning while enhancing discriminative ability, while maintaining model stability. From the accuracy metric perspective, the performance of various activation functions can be divided into three tiers: PReLU leads with an accuracy rate of 99.625%, forming the first tier; Mish and SiLU/Hardswish form the second tier with accuracy rates of 99.5% and 99.375%, respectively; while ReLU, ReLU6, and SELU are in the third tier with accuracy rates ranging from 98.125% to 98.75%. Notably, PReLU outperforms the baseline ReLU by 0.875 percentage points, an improvement that often translates to significant quality gains in practical applications. From the perspective of the loss function, PReLU’s loss value of 0.0183 is 22.6% lower than the next-best Mish (0.0235) and 66.9% lower than ReLU (0.0553). This substantial reduction in loss directly reflects the model’s stronger generalization ability. A deeper analysis of the characteristics of each activation function reveals that the best-performing PReLU and Mish both exhibit non-monotonicity, enabling them to better handle negative inputs and avoid the “neuron death” issue associated with the ReLU series. In contrast, the poorer-performing ReLU6 and SELU are limited in their expressive capabilities due to over-constraint or sensitivity. Notably, while Hardswish and SiLU belong to the Swish family of activation functions, their performance differs significantly, indicating that subtle mathematical form changes can lead to substantial performance differences. These experimental results provide important guidance for selecting activation functions in practical engineering applications, validate the superiority of PReLU in specific tasks, and also point to potential directions for future research.

### 3.5 Five-fold cross-validation results

As shown in **Table 5**, this study comprehensively evaluated the performance of the MobileNet-GDR model in grape leaf disease detection through five-fold cross-validation. The validation results fully demonstrate the model’s outstanding classification capability and excellent generalization performance. Across four core evaluation metrics, the model achieved an average accuracy of 99.39% in five-fold validation, with average precision, recall, and F1 score reaching 99.26%, 99.30%, and 99.29%, respectively. These figures indicate the model has attained near-perfect performance levels in grape leaf disease identification.

**Table 5:** Results of the five-fold cross-validation of the MobileNet-GDR.

Folding factor	Acc(%)	P(%)	R(%)	F1(%)
Fold-1	99.45	99.35	99.35	99.25
Fold-2	99.63	99.25	99.53	99.47
Fold-3	99.68	99.50	99.45	99.60
Fold-4	98.58	98.55	98.53	98.54
Fold-5	99.63	99.63	99.63	99.60

Specifically, the model’s high precision indicates an extremely low false positive rate during identification, effectively preventing healthy leaves from being misclassified as diseased. This is crucial for precise pesticide application and reducing pesticide misuse. Simultaneously, the high recall rate signifies an extremely low false negative rate, maximizing the detection of genuinely diseased leaves—essential for early disease warning and control. The high F1 score further confirms the model achieves an ideal balance between precision and recall, showcasing its outstanding overall performance.

Notably, in five-fold cross-validation, although metrics in Fold-4 showed slight fluctuations, all remained above 98.5%, while the other four folds demonstrated high consistency. This stability demonstrates the model’s strong adaptability to different data subsets and robust performance. Overall, the high performance and reliability of the MobileNet-GDR model in grape leaf disease detection make it highly valuable for practical agricultural applications.

### 3.6 The influence of parameter settings in group convolution and PReLU on the model results

**Figure 10:**
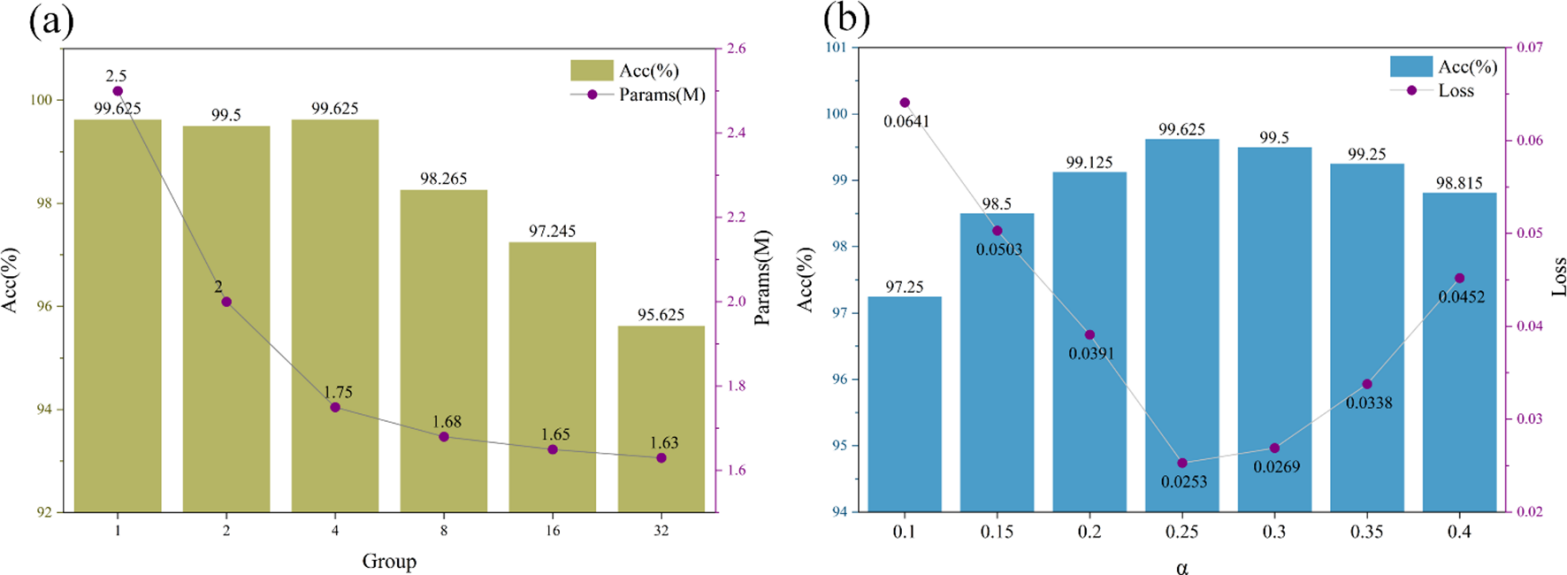
The influence of parameter settings in group convolution and PReLU on the model results. **(a)** The impact of the number of groups in grouped convolutions on model results, **(b)** The impact of parameter settings in PReLU on model results.

**Figure 10a** demonstrates the impact of group convolution configuration on model performance and complexity in grape leaf disease detection. The results reveal a critical trade-off between accuracy and parameter efficiency based on the number of groups. When increasing groups from 1 (standard convolution) to 4, the model maintains peak classification accuracy (99.625%) while reducing parameters from 2.50M to 1.75M, achieving a 30% reduction. This demonstrates that moderate grouping significantly enhances model efficiency without compromising performance, making it suitable for resource-constrained deployment environments. However, beyond 4 groups, model performance deteriorates substantially, with accuracy dropping from 99.625% to 95.625%. Excessive grouping disrupts feature interaction between channels, severely limiting the model’s representational capacity. Although parameter count further decreases to 1.63M, the accuracy loss becomes unacceptable for practical applications. Therefore, 4 groups represents the optimal configuration for this task, achieving the best balance between classification accuracy and model efficiency while maintaining practical applicability.

**Figure 10b** demonstrates the effect of PReLU parameter α on model performance. The results show that α=0.25 achieves optimal performance with 99.625% accuracy and 0.0253 loss. This value provides the best balance for feature learning and gradient propagation. When α increases beyond 0.25, both accuracy and loss deteriorate, indicating impaired learning capability. Conversely, lower α values (0.10-0.20) yield suboptimal results. These findings highlight the importance of proper activation function configuration for agricultural vision tasks. The study establishes α=0.25 as the optimal initialization for PReLU in grape leaf disease detection, providing guidance for similar agricultural applications. This configuration ensures effective model performance while maintaining training stability.

## 4 Discussion

The proposed MobileNet-GDR algorithm demonstrates significant advancements in lightweight grape leaf disease identification, achieving an exceptional classification accuracy of 99.625% while maintaining remarkably low computational requirements of just 1.75M parameters and 0.18G FLOPs. This outstanding performance positions our model as a highly effective solution for real-time agricultural applications, successfully addressing the critical challenge of balancing accuracy with efficiency in mobile deployment scenarios. The experimental results clearly indicate that MobileNet-GDR surpasses existing lightweight models, including FasterNet and GhostNet, in both diagnostic precision and computational efficiency, establishing its superiority in the field of mobile-based plant disease recognition.

The integration of depthwise separable convolutions with grouped convolutions forms the foundation of an exceptionally efficient feature extraction system, significantly reducing computational overhead while maintaining robust feature representation capabilities. This optimized approach to feature extraction is particularly crucial for identifying subtle disease patterns that may be easily overlooked in conventional models. Furthermore, the strategic incorporation of PReLU activation functions substantially improves the model’s nonlinear representation capacity compared to standard ReLU implementations, enabling more sophisticated pattern recognition for disease classification tasks. The redesigned feature fusion mechanism represents another critical advancement, as it effectively minimizes information loss during downsampling operations - a common limitation in many lightweight architectures that can severely impact performance on fine-grained classification tasks. This capability could revolutionize crop management practices by providing timely and accurate disease detection, ultimately leading to more targeted interventions and reduced pesticide usage.

A critical question arising from this work is how MobileNet-GDR conceptually and empirically distinguishes itself from other efficient architectures such as ShuffleNet and GhostNet, which also employ channel manipulation and enhanced non-linearity. Conceptually, ShuffleNet introduces channel shuffling to facilitate information flow between grouped convolutions, a mechanism primarily aimed at maintaining accuracy as network width increases. In contrast, our use of grouped convolution in MobileNet-GDR is more targeted: it serves as a direct and parameter-efficient replacement for the computationally expensive pointwise convolutions in MobileNetV4, forming a leaner base. More importantly, we pair this with PReLU not as a generic non-linearity, but explicitly to amplify the model’s sensitivity to the low-intensity, high-frequency textural patterns that characterize early-stage disease spots. This design philosophy is inherently task-specific, prioritizing feature quality for fine-grained pathology over generic classification. While ShuffleNet and GhostNet are formidable competitors on general benchmarks, MobileNet-GDR consistently achieves superior accuracy under comparable or lower computational budgets on our grape disease dataset. We attribute this to the fact that our model’s inductive biases are more aligned with the demands of the task. The PReLU’s adaptive gradients appear better suited for capturing the nuanced visual cues of plant diseases than the fixed operations in ShuffleNet or the linear transformations in GhostNet modules.

Despite the promising results, this study is not without its limitations, which also present avenues for future work. A primary consideration is the generalizability of the model. The dataset used for training and validation, while substantial, was acquired under a specific set of conditions regarding lighting, leaf orientation, and background. Consequently, the model’s performance may be susceptible to variations encountered in truly uncontrolled field environments, such as different times of day, weather conditions, or occlusions by other plant parts. This potential dataset bias warrants further investigation. Furthermore, while our proposed architectural modifications have demonstrated efficacy in terms of accuracy and computational efficiency, their practical deployment on resource-constrained edge devices remains to be fully quantified. The reported high inference speed was achieved on a high-performance computing server. A more rigorous validation involving benchmarking on a range of actual mobile and embedded hardware (e.g., smartphones, Raspberry Pi, or Jetson Nano) is necessary to conclusively support our claims of real-time capability in practical applications. Finally, although Grad-CAM visualizations suggest that our model focuses on semantically relevant regions, a more rigorous, quantitative assessment of model interpretability is lacking. The current evaluation is primarily qualitative. Future work would benefit from engaging domain experts to systematically evaluate the clinical relevance of the model’s explanations or by employing quantitative metrics for interpretability, which is crucial for building trust with end-users in agricultural settings.

## 5 Conclusions

This paper addresses the practical needs of grape leaf disease diagnosis by proposing a lightweight image classification algorithm, MobileNet-GDR. Through careful design of the network architecture and optimization of training strategies, the algorithm achieves high accuracy while significantly improving computational efficiency. Compared to existing mainstream lightweight models, MobileNet-GDR achieves over 20% higher computational efficiency while maintaining comparable accuracy. The significance of this study lies not only in proposing an efficient grape disease classification algorithm but also in exploring optimization paths for lightweight CNNs in agricultural intelligence.

Future research will focus on expanding the model’s capabilities through cross-crop disease transfer learning, investigating its generalizability across different plant species and disease types. Concurrently, we plan to develop user-friendly mobile applications to bridge the gap between theoretical research and practical implementation, facilitating seamless technology transfer to agricultural stakeholders. This deep integration of advanced deep learning techniques with modern agricultural practices is expected to revolutionize disease management strategies by enabling: (1) early disease detection through real-time field diagnosis, (2) data-driven decision-making for precision pesticide application, and (3) large-scale crop health monitoring systems. Such technological advancements will contribute significantly to sustainable agricultural development by reducing chemical inputs, minimizing crop losses, and optimizing resource allocation in precision farming systems.

## Data Availability

The original contributions presented in the study are included in the article/supplementary material. Further inquiries can be directed to the corresponding author.
